# Carbon Monoxide (CO), Nitric Oxide, and Hydrogen Sulfide Signaling During Acute CO Poisoning

**DOI:** 10.3389/fphar.2021.830241

**Published:** 2022-03-18

**Authors:** Ronald F. Coburn

**Affiliations:** Department of Physiology, The Perelman School of Medicine, University of Pennsylvania, Philadelphia, PA, United States

**Keywords:** carbon monoxide poisoning, redox signaling, carbon monoxide signaling, nitric oxide signaling, hydrogen sulfide signaling, gaso-transmitters

## Abstract

Major toxic effects of acute carbon monoxide (CO) poisoning result from increases in reactive oxygen species (ROS) and reactive nitrogen species (RNS) producing oxidative stress. The importance of altered nitric oxide (NO) signaling in evoking increases in RNS during CO poisoning has been established. Although there is extensive literature describing NO and hydrogen sulfide (H_2_S) signaling in different types of cells under normal conditions, how CO poisoning-evoked deregulation of additional NO signaling pathways and H_2_S signaling pathways could result in cell injury has not been previously considered in detail. The goal of this article was to do this. The approach was to use published data to describe signaling pathways driven by CO bonding to different ferroproteins and then to collate data that describe NO and H_2_S signaling pathways that could interact with CO signaling pathways and be important during CO poisoning. Arteriolar smooth muscle cells—endothelial cells located in the coronary and some cerebral circulations—were used as a model to illustrate major signaling pathways driven by CO bonding to different ferroproteins. The results were consistent with the concept that multiple deregulated and interacting NO and H_2_S signaling pathways can be involved in producing cell injury evoked during acute CO poisoning and that these pathways interact with CO signaling pathways.

## Introduction

Recent reviews of the pathogenesis of the toxicity resulting from acute carbon monoxide (CO) poisoning have emphasized increases in reactive oxygen species (ROS) and reactive nitrogen species (RNS) that evoke oxidative stress (Piantadosi, 2008; Akyol et al., 2014; Roderique et al., 2015). Other articles describe roles of ion channels (Peers, 2011), impaired metabolism–blood flow coupling (M-BFC) and alveolar ventilation–blood flow coupling (Coburn, 2020), tissue hypoxia (Darling and Roughton, 1944), increased carboxymyoglobin % saturation ([COMb]) (Sangali and Bidanset, 1990), and increased free heme (Cronje et al., 2004). The goal of the present article is to consider using previously published data if nitric oxide (NO) and hydrogen sulfide (H_2_S) signaling pathways could interact with CO signaling pathways during acute CO poisoning. The importance of altered NO signaling in evoking increases in reactive nitrogen species (RNS) during CO poisoning has been established (Ischiropoulos et al., 1996; Thom et al., 1997). Roderique et al. (2015) emphasize the importance of NO signaling during CO poisoning. However, it has not been previously considered in detail if additional NO signaling could be a factor in cell injuries that occur under this condition. In addition, the possible roles of H_2_S signaling during CO poisoning have not been addressed in previous CO poisoning reviews. There is extensive literature, some of which are cited later in this article, describing normal CO, NO, and H_2_S signaling in different types of cells and the cross-talk signaling that occurs between the three gaso-transmitters. The reader is referred to publications by Treuer and Gonzalez (2015) and Radi (2018) who reviewed some general properties of NO signaling mechanisms and by Li et al. (2011), Paul and Snyder (2014), Kabil et al. (2014), and Giuffre and Vincent (2018) who reviewed the properties of normal H_2_S signaling. An important review has been published of relationships between CO and NO signaling and interactions between the heme oxygenase and nitric oxide synthase systems oriented to normal signaling (Wu and Wang, 2005).

For this analysis, a vascular model was selected to illustrate major signaling pathways driven by CO bonding to different ferroproteins (FPs). Smooth muscle cells (SMCs) with neighboring endothelial cells (ECs) located in arterioles in the coronary and some cerebral circulations were chosen for this model because there is some available information regarding the FPs that reside within these cells (Coburn, 2020). In addition, there is evidence that NO and H_2_S are involved when M-BFC in these circulations is impaired during CO poisoning (Coburn, 2020). With the model, NO formed in ECs is transported into SMCs. This transport may not be explained by simple diffusion. In some small arteries, monomeric hemoglobin (Hb) *a* expressed in endothelial cells and enriched at the myoendothelial junction can regulate eNOS and NO transport into SMCs (Straub et al., 2012). A major function of the arteriolar SMCs and ECs is to affect M-BFC, also called metabolic vasodilation, which is defined as coupling between O_2_ consumption and blood flow. This mechanism promotes PO_2_ uniformity within tissues, that is, prevents regional hypoxia, at least partially by controlling capillary recruitment and capillary densities. O_2_ sensors located within both arteriolar SMCs and partner ECs sense low PO_2_ in tissue areas that have high O_2_ uptake/blood flow ratios which evokes SMC relaxations producing vasodilation (Coburn, 2020). M-BFC impairment in these circulations during CO poisoning was considered to be due to the loss of arteriolar SMCs and EC O_2_ sensing, unregulated endothelial NO formation, effects on ion channels, and increases in ROS signaling (Coburn, 2020)**.**

As indicated before, the present article is organized around CO bonding to FPs (Caughey, 1970; Traylor and Berzinis, 1980; Coburn, 2021). The reason for this, as stated before, is that this bonding activates or inactivates signaling pathways. This approach also facilitated comparisons of signaling pathways driven by CO bonding to FPs, and NO and H_2_S signaling pathways. Approaching CO poisoning by considering CO bonding to FPs follows the “surprising” non-reactivity of CO in mammalian tissues (Piantadosi, 2008) and that CO bonding to FPs usually occurs in their Fe^2+^ or Fe^3+^ oxidative states. It is possible that limiting CO signaling pathways to those initiated by CO bonding with FPs is missing some signals operating during CO poisoning. FPs considered in the SMC–EC duet in this study include cytochrome C oxidase (COX), NADPH oxidase (NOX), myoglobin (Mb), endothelial NO synthase (eNOS), catalase and peroxidase, cystathionine *ß* synthase (CBS), prolyl hydroxylase–hypoxia-induced factor (PHD), heme oxygenase-2 (HO-2), the K^+^ ion channels described below, and EC monomeric Hb *a*. These FPs include hemoproteins and proteins that require binding of a prosthetic heme for their functions, or like PHD contain non-heme iron. HO-2 has heme-binding sites (Wu and Wang, 2005). CBS activity is dependent on the presence of free hemes (Taoka and Banerjee, 2001; Banerjee, 2017). Heme is involved in CO activation–inactivation mechanisms in K_ATP_, BK_Ca_, and Kv channels (Horrigan et al., 2005; Jaggar et al., 2005; Hou et al., 2009; Sahoo et al., 2013; Burton et al., 2016). Evidence that free hemes can increase during CO poisoning is cited later. However, the importance of levels of free hemes in controlling the operation of these CO targets during CO toxicity scenarios is not known. It is possible that CO evokes changes in BK_Ca_ channel activity by a mechanism independent of CO binding to its prosthetic heme (Hou et al., 2009).

Possible FP CO targets involved in evoking cell injury during acute CO poisoning considered in this article included some that were not, to the best of the author’s knowledge, described in previous CO poisoning reviews. These include the HO-2 and PHD O_2_ sensors, ion channels involved in M-BFC, and EC monomeric Hb *a*. There is kinetic evidence that HO-2 activity can be by-product-inhibited during CO poisoning (Matsui et al., 2010). *In vivo* evidence was obtained by showing large decreases in endogenously formed CO during Hb catabolism in dogs after [COHb] was increased via CO inhalation (Coburn et al., 1967). Evidence that the O_2_ sensing function of PHD can be inhibited by CO was provided by Goldberg et al. (1988), Huang et al. (1999), Hagen et al. (2003), and Mbenza et al. (2021). Evidence that increases in P_CO_ can alter the ion channel function and O_2_ sensors that affect M-BFC has been published (Coburn, 2020). Of the arteriolar SMC-EC CO targets listed before, only mitochondrial and NOX–ROS formations are proven to evoke cell injury during CO poisoning. Mb, Hb, eNOS, K^+^ channels, and catalase are likely important CO targets during CO poisoning scenarios. HO-2, PHD, CBS, and EC monomeric Hb *a* are possible targets.

The literature search was limited to posttranslational signaling. It did not cover downstream cellular toxicity mechanisms resulting from increased [ROS], [RNS], or hypoxia; CO activation of different kinases and soluble guanylyl cyclase; NO signaling to eicosanoids; or signaling resulting from effects of the three gaso-transmitters on oxidative phosphorylation. The analysis given in this article has not been extended to include the discussion of complex relationships between tissue hypoxia and increased tissue P_CO_ in the pathogenesis of acute CO poisoning-evoked cell injury.

## Possible FP CO targets during CO poisoning: possible interactions between CO, NO, and H_2_S signaling pathways

The following section of this article lists the FP CO targets in the SMC-EC model and in blood to illustrate how CO, NO, and H_2_S signaling could be altered or deregulated during CO poisoning scenarios. Some characteristics of these FPs relevant to CO poisoning are described.

***BK*_*Ca*_, *K*_*ATP*_, *Kv*_*1.5*_, *and L-type Ca* ^*2+*^ *ion channels*:** As stated before, there is evidence that these arteriolar SMC ion channels are involved in CO-evoked impairment of M-BFC in coronary and some cerebral circulations (Coburn, 2020). In the cerebral circulation, H_2_S-evoked opening of K_ATP_ and opening or closing BK_Ca_ channels may be involved (Hou et al., 2009; Li et al., 2011; Morikawa et al., 2012; Coburn, 2020). CO inhibitory effects on L-type Ca^2+^ channels are indirect, mediated by increased [ROS] (Scragg et al., 2008). CO-evoked effects on other ion channels are not discussed in this article because they are not relevant to what we presently know about the arteriolar SMC-EC model.

***Catalase and peroxidases*:** These enzymes which scavenge ROS are inhibited by CO, a result of CO binding to their prosthetic hemes. CO ligation mechanisms differ with catalases and peroxidases and occur at different iron oxidative states during catalysis (Carlsson et al., 2005). Catalases are likely more important as ROS scavengers because of their rapid reaction rates with H_2_O_2_ (Carlsson et al., 2005). Important to the goals of this article, NO also inhibits catalase (Purwar et al., 2011). The ability of these proteins to scavenge ROS and exert some control of the cellular oxidant–antioxidant balance could be impaired during CO poisoning.

***CBS:*** The effects of CO poisoning on H_2_S signaling could be a result of inhibition of CBS, which results in a decreased formation of H_2_S (Puranik et al., 2006; Paul and Snyder, 2015; Banerjee, 2017). The dependency of this reaction on the presence of free heme is described before. H_2_S can scavenge ROS and RNS and is a powerful antioxidant considered to be critical for the intracellular balance between oxidants and antioxidants (Shefa et al., 2018). As described before, H_2_S-evoked effects on K_ATP_ channels are likely to be important in CO poisoning-evoked impairment of M-BFC. CBS is inhibited by NO **(** Taoka and Banerjee, 2001), a property relevant to NO signaling during CO poisoning.

*COX*, *NOX*, *and xanthine oxidase:* CO bonding to COX and resulting ROS generation within mitochondrial complexes I and III are currently thought to be the major cause of acute CO poisoning-evoked cell injury (Zuckerbraun et al., 2007; Akyol et al., 2014; Almeida et al., 2015; Roderique et al., 2015; Motterlini and Foresti, 2017). Increased P_CO_ can evoke ROS formation via NOX that results in cell injury (Kietzmann and Gorlach, 2005; Manea, 2010; Hara et al., 2017). Xanthine oxidase can be involved in CO poisoning-evoked ROS formation (Thom, 1992).

***eNOS:*** This hemoprotein, which normally catalyzes the formation of NO in ECs, can be inhibited during CO poisoning (Thorup et al., 1999; Hara et al., 2003; Chen and Meyrick, 2004; Choi and Kim, 2021). (This is contrasted with increases in the eNOS activity that occur during exposure to low [CO] (Thorup et al., 1999).) Increased NO formation during CO poisoning (Ischiropoulos et al., 1996; Thom et al., 1997) is likely due to CO displacement of NO bound to FPs (Thorup et al., 1999; Di Meo et al., 2016). Brain toxicity during CO poisoning can be mediated by NO (Meilin et al., 1996). The rapid reaction of NO with ROS results in formation of RNS, including peroxynitrite and other oxidative NO by-products, major causes of cell injury during CO poisoning (Radi, 2018). NO is a free radical that is a strong oxidant that can cause cell injury during CO poisoning. As indicated before, increased or deregulated [NO] can impair coronary and cerebral M-BFC during CO poisoning (Coburn, 2020). Increased [NO] can scavenge H_2_S (Taoka and Banerjee, 2001). NO can inhibit CBS, resulting in decreased H_2_S formation (Tang et al., 2013) which in turn can cause increased NO formation (Mazza et al., 2013; Olas, 2015). Uncoupling of eNOS can produce ROS (Karbach et al., 2014). NO can inhibit HO-2 (Ding et al., 1999; Wu and Wang, 2005). The relationship of EC monomeric Hb *a* and eNOS formed NO is cited later.

***Hemoglobin (Hb)*:** In blood: Increases in [COHb] evoke oxyhemoglobin dissociation curve shifts which decrease mean capillary PO_2_ (PcO_2_) producing tissue hypoxia (Darling and Roughton, 1944). Increased [COHb]-evoked decreases in deoxyHb likely inhibit the Hb-nitrite reductase activity decreasing NO formation and may inhibit Hb NO scavenging (Kim-Shapiro et al., 2006). Endothelial monomeric Hb α: In some resistance arteries, endothelial monomeric ferrous Hb *a*-evoked increases in NO signaling to SMCs can be increased three fold by CO exposure (Straub et al. (2012). Thus, this mechanism could contribute to increase in [NO] that can occur during CO poisoning.

***HO-2*:** As cited before, there is evidence that the HO-2 activity can be inhibited during CO poisoning. Effects relevant to CO poisoning include 1) that due to inhibition of its function as an O_2_ sensor (Kemp, 2005) involved in CO-evoked impairment of M-BFC discussed before; 2) that due to inhibition of its function as a scavenger of free heme (Kumar and Bandyopadhyay, 2005; Chiabrando et al., 2014) following evidence that levels of free heme can increase markedly during severe CO poisoning (Cronje et al., 2004); and 3) that resulting from decreases in the formation of biliverdin/bilirubin which are important antioxidants and may scavenge ROS via biliverdin reductase cycling (Janson and Daiber, 2012). In addition to CO-evoked effects, the HO-2 O_2_ sensor can be inhibited by H_2_S (Matsui et al., 2018). As cited before, NO can bind to the heme moiety of HO-2 and inhibit its function (Ding et al., 1999; Wu and Wang, 2005). In piglet cerebral microvessels, glutamate-stimulated NOS produced NO that evoked CO formation via HO-2 (Leffler et al., 2005).

***Mb:*** Effects of increased [COMb] are 1) inhibition of Mb-facilitated delivery of O_2_ to oxidases and oxygenases (Sangali and Bidanset, 1990); 2) decreases in the ability of Mb to scavenge NO (Flogel et al., 2001); and 3) the inhibition of Mb-nitrite reductase (Hendgen-Cotta et al., 2014).

***PHD:*** Huang et al. (1999) and Zepeda et al. (2013) have reviewed the normal roles of PHD which functions as an O_2_ sensor that regulates the transcription factor HIF-1α which, in turn, upregulates multiple genes involved in adapting to hypoxia. This PHD senses hypoxia which results in a decrease in HIF-1 degradation, that is, stabilization of HIF-1α. Although during normoxia low [CO] augmented HIF-1α stabilization (Chin et al., 2007), as stated before several investigators have demonstrated that CO can inhibit PHD O_2_ sensing and suppress the activation of the HIF-1α-mediated response to hypoxia (Goldberg, et al., 1988; Huang et al., 1999; Hagen et al., 2003; Mbenza et al., 2021). NO can also impair PHD O_2_ sensing and hypoxia-induced HIF-1α stabilization (Huang et al., 1999; Brune and Zhou, 2007). Both mitochondrial- and NOX-derived ROS can inhibit PHD O_2_ sensing (Kietzmann and Gorlach, 2005; Zepeda et al., 2013). CO-evoked impairment of its O_2_ sensing function could result in regional hypoxia (Hagen et al., 2003; Zepeda et al., 2013) or augment tissue hypoxia resulting from increased [COMb] or [COHb], or impairment of M-BFC.

### Signaling Pathways

**FIGURE 1:**
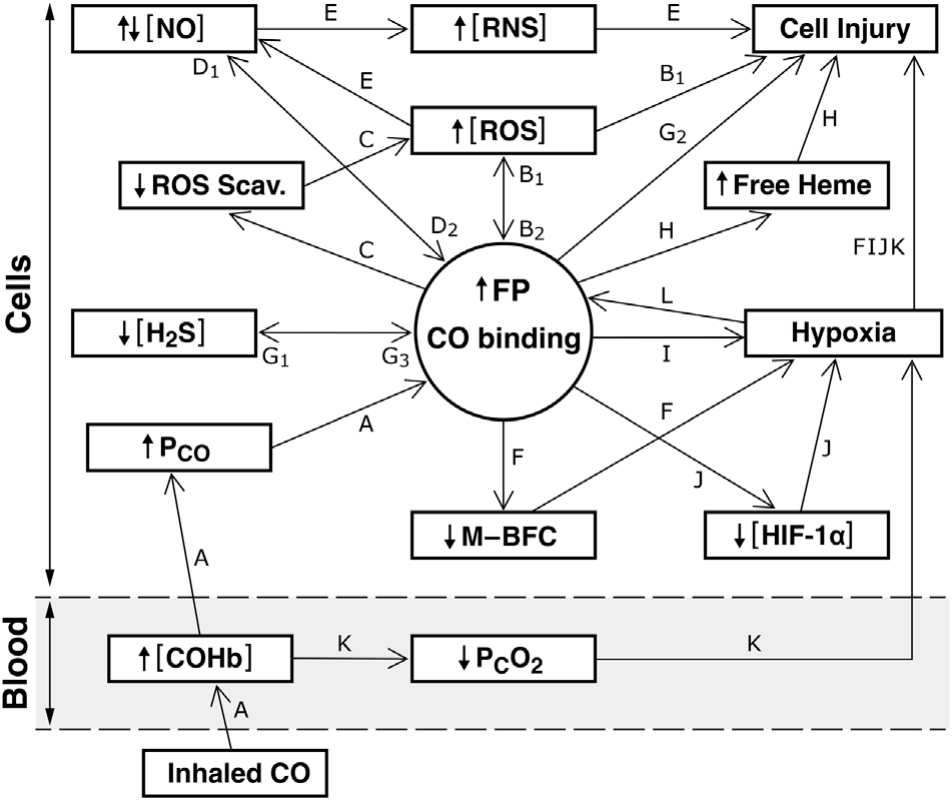
Possible interactions between CO, NO, and H_2_S signaling pathways in an arteriolar SMCs-ECs model. The schema drawn above the dotted line indicates intracellular reactions (within SMCs and ECs). Below the interrupted line are the events in blood in the coronary or cerebral circulations. Because the goal of this article was to highlight effects of CO bonding with FPs, this is depicted as a central circle. FPs considered here are described in the text. Arrows illustrate the different signaling pathways possibly involved in CO poisoning-evoked cell injury. The various signaling pathways are described in the text. Abbreviations: CO, carbon monoxide; NO, nitric oxide; H_2_S, hydrogen sulfide; ROS, reactive oxygen species; RNS, reactive nitrogen species; M–BFC–metabolism, blood flow coupling; HIF-1α, hypoxia-induced factor-1α; PcO_2_, mean capillary PO_2_; ROS Scav, ROS scavengers.

Figure 1 depicts possible signaling pathways described before. [NO] and [H_2_S] indicate bioactivities. Pathways i to xiv consider CO signaling and pathways xv to xxvi emphasize NO and H_2_S signaling.

i) Pathway A: Inhaled CO → ↑ [COHb] → ↑ tissue P_CO_ → CO binding to FPs (Coburn, 2021). Increased tissue P_CO_ at a given [COHb] is a result of the rapid reaction of CO + O_2_Hb so that blood entering peripheral tissues is in chemical equilibrium with the [COHb] [O_2_Hb] and PO_2._ Because CO diffuses as efficiently as O_2_, the steady state means P_CO_ in gas exchange arterioles and capillaries is considered equal to the mean tissue P_CO_ (Coburn, 2017).
ii) Pathways A → B1: ↑ tissue P_CO_ → CO binding to COX and/or NOX → ↑ [ROS] → cell injury (Zuckerbraun et al., 2007; Manea, 2010; Akyol et al., 2014; Almeida et al., 2015; Roderique et al., 2015; Choi and Kim, 2021).
iii) Pathway A → B1: CO activation of xanthine oxidase → ↑ [ROS] → cell injury (Thom, 1992).
iv) Pathways A → B1 → B2 → F: CO binding to COX or NOX → ↑ (deregulated) [ROS] → impaired M–BFC → regional tissue hypoxia (Coburn, 2020).
v) Pathways A → F: CO inhibition of HO-2 O_2_ sensing → impaired M–BFC → regional hypoxia (Coburn, 2020).
vi) Pathways A → F: CO effects on K_ATP_, BK_Ca_, Kv_1.5_ channels → impaired M-BFC (Horrigan et al., 2005; Jaggar et al., 2005; Hou et al., 2009; Burton et al., 2016). CO inhibition of L-type Ca2+ channels is indirect, due to ↑ ROS (Scragg et al., 2008).
vii) Pathways A → C → last segment of B1: CO binding to catalases and peroxidases inhibits these enzymes → ↑ [ROS] → cell injury (Carlsson et al., 2005).
viii) Pathways A → C → last segment of B1: CO inhibition of HO-2 → ↓ biliverdin/bilirubin formation and biliverdin reductase-evoked scavenging of ROS → ↑ [ROS] → cell injury (Janson and Daiber, 2012).
ix) Pathways A → H: CO-evoked ↓ HO-2 activity → ↓ heme scavenging → ↑ free heme → cell injury (Kumar and Bandyopadhyay, 2005; Chiabrando et al., 2014). In addition, increased free heme may modify the function of ion channels dependent on prosthetic heme binding (see Pathway vi.)
x) Pathways A → I: CO binding to Mb → ↑ COMb → tissue hypoxia (Sangali and Bidanset, 1990).
xi) Pathways A → J: CO binding to PHD → ↓ hypoxia activation that is, loss of its O_2_ sensing function → tissue hypoxia (Huang et al., 1999; Hagen et al., 2003).
xii) Pathways A → B1 → B2 → J: ↑ in mitochondrial- and NOX-generated [ROS] → ↓ the ability of PHD to sense PO_2_ → global tissue hypoxia and possibly regional hypoxia (Zepeda et al., 2013).
xiii) The first segment of pathway A followed by Pathway K: ↑ [COHb] → ↓ mean capillary PO_2_ → tissue hypoxia (Darling and Roughton, 1944). Not shown - that the ability of Hb to scavenge NO is inhibited by ↑ [COHb]-evoked decrease in deoxyHb (Kim-Shapiro et al., 2006).
xiv) Pathway L: Cellular hypoxia evokes ↑ CO binding to unidentified FPs which could facilitate other pathways driven by CO binding to FPs (Coburn, 2021).
xv) Pathways A → D1: CO displaces NO from its binding to FPs → ↑ [NO] (Thorup et al., 1999; Di Meo, et al., 2016). Normal mechanisms that increase NO formation, eNOS and Mb nitrite reductase, can be inhibited during CO poisoning (Chen and Meyrick, 2004; Hendgen-Cotta et al., 2014).
xvi) Pathways A → D1: CO increases NO transport from EC to SMC via binding to the ferrous oxidative state of EC monomeric Hb *a* → ↑ [NO] (Straub et al., 2012). (This could occur under conditions where eNOS is not completely inhibited.)
xvii) Pathways A → B1 → ROS + NO → E: [NO] + ↑ ROS → ↑ [RNS] → cell injury. (Ischiropoulos et al., 1996; Thom et al., 1997). NO, a free radical, is a powerful oxidant and cell injury results from increases in [NO] as well as [RNS] (Radi, 2018). Whether [NO] increases or decreases depends on its rate of formation versus its rate of conversion to RNS.
xviii) Pathways A → G1 → G3 → G2 and B1: CO binding to CBS → ↓ [H_2_S] → cell injury due to a ↓ in its antioxidant action and ↓ H_2_S scavenging of ROS → ↑ [ROS] (Puranik et al., 2006; Paul and Snyder, 2015; Giuffre and Vincent, 2018).
xix) Pathways A → D1 → D2 → G1: ↑ [NO] → ↓ CBS → ↓ [H_2_S] (Taoka and Banerjee, 2001; Tang et al., 2013). Possible effects of a ↓ [H_2_S] are outlined before.
xx) Pathways A → D1 → D2 → C → the last segment of B1: ↑ [NO] → ↓ HO-2 activity → ↓ biliverdin/bilirubin ROS scavenging → ↑ [ROS] and ↓ heme scavenging (Ding et al., 1999; Wu and Wang, 2005). This pathway also depicts that ↑ [NO] → ↓ catalase activity → ↑ [ROS] (Purwar et al., 2011).
xxi) Pathways A → D1 → D2 → F: ↑ or deregulated [NO] → impaired M-BFC → regional tissue hypoxia (Coburn, 2020).
xxii) Pathways A → G1 → G3 → B1: ↓ H_2_S binding to COX → ↑ [ROS] (Kabil et al., 2013).
xxiii) Pathways A → G1 → G3 → D1 → E → ↑ [RNS]: ↓ [H_2_S] → ↑ [NO] → ↑ [RNS] (Mazza et al., 2013; Olas, 2015).
xxiv) Pathways A → G1 → G3 → F: CO bonding to CBS → ↓ [H_2_S] → impaired M–BFC (Coburn, 2020). Other possible effects of ↓ [H_2_S] are described before.
xxv) Pathways A → D1: CO binding to Mb → ↑ [COMb] → ↓ Mb NO scavenging → ↑ [NO] (Flogel et al., 2001) → deleterious effects are described before.
xxvi) Pathways A → D1 → D2 → J: ↑ [NO] → loss of PHD O_2_-sensing → global or regional tissue hypoxia (Huang et al., 1999; Zepeda et al., 2013).

## Discussion

Using published data, the question is asked if NO and H_2_S signaling could interact with CO signaling pathways and be a factor in CO poisoning-evoked cellular injury. The approach of describing CO poisoning from the viewpoint of CO binding to FPs that exerts control of various signaling pathways illustrates the spectra of different CO targets considered in this article. For this analysis, a vascular model was used to illustrate major signaling pathways driven by CO bonding to different FPs. Other cells contain many of the same FPs. Thus, some of the possible NO and H_2_S signaling pathways and interactions with CO signaling pathways described using this model are projected to be valid for other cell types. As indicated in the introduction, the importance of CO binding to many of the FPs considered in this article as a function of different levels of CO poisoning is not known.

In this article, the well-known effects of increased [NO] that occur during CO poisoning, described in pathways xv and xvii, were expanded to include NO-evoked inhibition of CBS-evoked H_2_S formation, impairment of M-BFC, inhibition of O_2_ sensing by PHD, inhibition of HO-2 free heme and ROS scavenging, and inhibition of catalase. Several reactions are described that could exert positive feedback effects on NO formation during CO poisoning (pathways xvi, xxiii, and xxv) augmenting Pathway xv evoked increased [NO]. This article also emphasizes possible CO poisoning-evoked changes in H_2_S signaling where effects of a decreased [H_2_S] include impaired ROS scavenging and suppression of its function as an antioxidant, and impaired M-BFC. Possible interactions between NO, H_2_S, and CO signaling that could be important during CO poisoning are described. These include that both CO and NO bonding with CBS can inhibit H_2_S formation; that a decreased [H_2_S] can augment [NO] increases; that both increased P_CO_ and increased [NO] (as well as increased [ROS] and [RNS]) can impair the PHD function as an O_2_ sensor; that both increased P_CO_-evoked [H_2_S] decreases and [NO] changes can impair M-BFC; that NO and CO can inhibit catalase activity; and that NO and H_2_S as well as CO can inhibit HO-2 activity. Cited data indicate interactions between H_2_S, NO, and ROS signaling. These include that both H_2_S and NO can be involved in mediating [ROS] or [RNS]. ROS targets considered in this article include PHD and multiple ion channels involved in M–BFC. Thus, it is suggested that alterations in NO and H_2_S signaling during CO poisoning scenarios may occur in tandem with deregulation of ROS signaling. Findings also suggest that normal NO and H_2_S signaling pathways, like normal endogenous CO signaling, may be deregulated during CO poisoning. This would not just be a result of changes in bioactivities of the gaso-transmitters described before. Precise signaling at various cellular and subcellular targets is likely lost as is well recognized to occur during increased P_CO_-evoked redox signaling. Whether deregulated NO and H_2_S signaling could contribute to cell injury occurring during different levels of CO poisoning is another issue. An argument supporting this hypothesis is that multiple deregulated and interacting NO and H_2_S signaling pathways are likely to be involved in producing or mediating cell injury evoked during CO poisoning because these pathways can potentially interact with CO signaling pathways that trigger cell injury.

Figure 1 and the previous discussion do not consider the relative importance of the different NO and H_2_S signaling pathways as effectors of cell injury during CO poisoning scenarios. It is not possible yet to describe a mechanistic continuum of the different pathways. It seems possible that cell injury resulting from different severities or time durations of CO poisoning involves different segments of a signaling network. However, because of a lack of NO and H_2_S binding and bioactivity information, it is not established that any of the NO pathways described before, other than those shown in pathways xv and xvii, nor any of the H_2_S pathways, operate in the SMC-EC model during CO poisoning. A weakness in the approach used in constructing the pathways shown in the Figure is that data taken from published results were obtained using different types of cells, and none of them include arteriolar SMC-ECs. In addition, the discussion of H_2_S pathways does not consider that metabolic pathways other than CBS can form H_2_S, or effects on H_2_S degradation during CO poisoning. There are also issues related to subcellular locations where H_2_S is formed. It is not clear how multiple downstream H_2_S reactions could influence [H_2_S]-dependent mechanisms described in this article. A major emphasis in this article is on H_2_S functions as an antioxidant and ROS scavenger during oxidative stress and its role in M-BFC. For NO, this article only considers reactions related to CO poisoning, on top of large literature describing multiple functions of NO signaling. However, even considering these caveats, it seems justified to conclude that listing and describing possible NO and H_2_S signaling pathways as described in this study is a step forward toward understanding how signaling of these gaso-transmitters could function during acute CO poisoning scenarios. More research is necessary to better delineate the importance and details of deregulated NO signaling and H_2_S signaling during CO poisoning-evoked cell injury. As stated before, more binding and NO and H_2_S bioactivity data are needed. The importance of specific FP CO targets in generating signaling also depends on their CO binding constants, and a challenge for future research is to determine these constants and the extent of CO bonding to different FPs at different P_CO_. There are calculated data that suggest that tissue P_CO_ in resting humans could increase 50- to 60-fold as the [COHb] increases from normal to 40% saturation (Coburn, 2017). There also is evidence, so far limited to red skeletal muscle and heart muscle, that extravascular CO binding increases as [COHb] increases (Coburn, 2021).

Whether or not NO and H_2_S signaling could interact with CO signaling during CO poisoning-evoked tissue hypoxia has not been addressed in detail in this article. However, signaling pathways depicted in the Figure show multiple interactions that could occur in the arteriolar SMC-EC model. Increased P_CO_-evoked impairment of M-BFC can result in regional tissue hypoxia. Increased P_CO_-evoked impairment of PHD-[HIF-1α] can evoke regional or global hypoxia. Increased [COMb] can inhibit intracellular O_2_ delivery, and increased [COHb] can evoke tissue hypoxia due to shifts in the oxyhemoglobin dissociation curve. Increased P_CO_-evoked impairment of alveolar–ventilation–blood flow coupling can cause decreases in PaO_2_ and tissue hypoxia (Coburn, 2020), which is not shown in the Figure. Increased cellular P_CO_ can evoke cellular and tissue hypoxia via several different mechanisms listed before, and cellular hypoxia can result in increased CO binding to FPs, emphasizing that multiple interactions between tissue hypoxia and increased P_CO_ can occur during CO poisoning scenarios.

## Conclusion

i) This study explores how multiple NO and H_2_S signaling pathways could interact with CO signaling pathways during acute CO poisoning scenarios. An arteriolar SMC–EC duet was used as a model in this analysis. Some of the signaling pathways operating in this model likely occur in other cells that have some of the same FPs.
ii) Results are consistent with the hypothesis that during acute CO poisoning scenarios, multiple deregulated and interacting NO and H_2_S signaling pathways interact with deregulated CO and ROS signaling pathways.
